# Supplementary material for: Medical, socioeconomic, and geographic disparities in primary health care access and utilization: a population-based study of 8038 individuals aged one year and older in rural Uganda
Source: BMC Med. 2026 Jul 21;24:569. doi: 10.1186/s12916-026-05061-x (PMC13682141; doi:10.1186/s12916-026-05061-x)
Supplement: Supplementary file 1 [file 12916_2026_5061_MOESM1_ESM.pdf]

Supplementary information

Additional file 1: Figures S1-S3, Tables S1-S7. Fig S1: Histogram of HH distances to PHCs and medicine shops. Fig S2: Model for any care sought for adults. Figure S3: Model for type of care sought for adults. Table S1: Self-reported medical history. Table S2: Prevalence of past fever and diarrhoea among symptomatic participants across the study districts. Table S3: Care-seeking behaviour across study districts. Table S4: HH characteristics. Table S5: Characteristics of adults. Table S6: HH distances to PHCs and medicine shops by district. Table S7: Reported final diagnoses from government health centres.

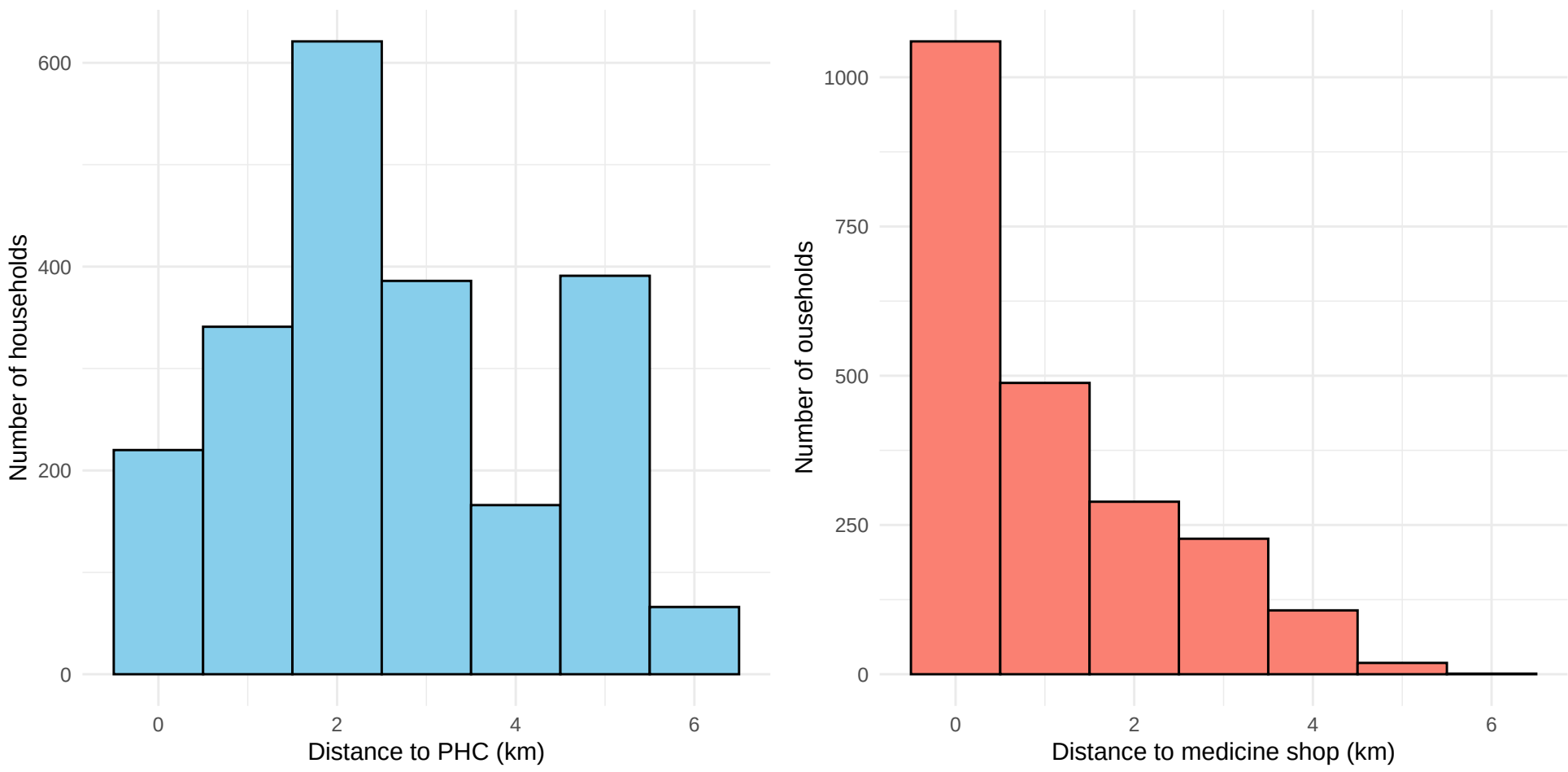

Fig. S1 Histogram of HH distances to PHCs and medicine shops.

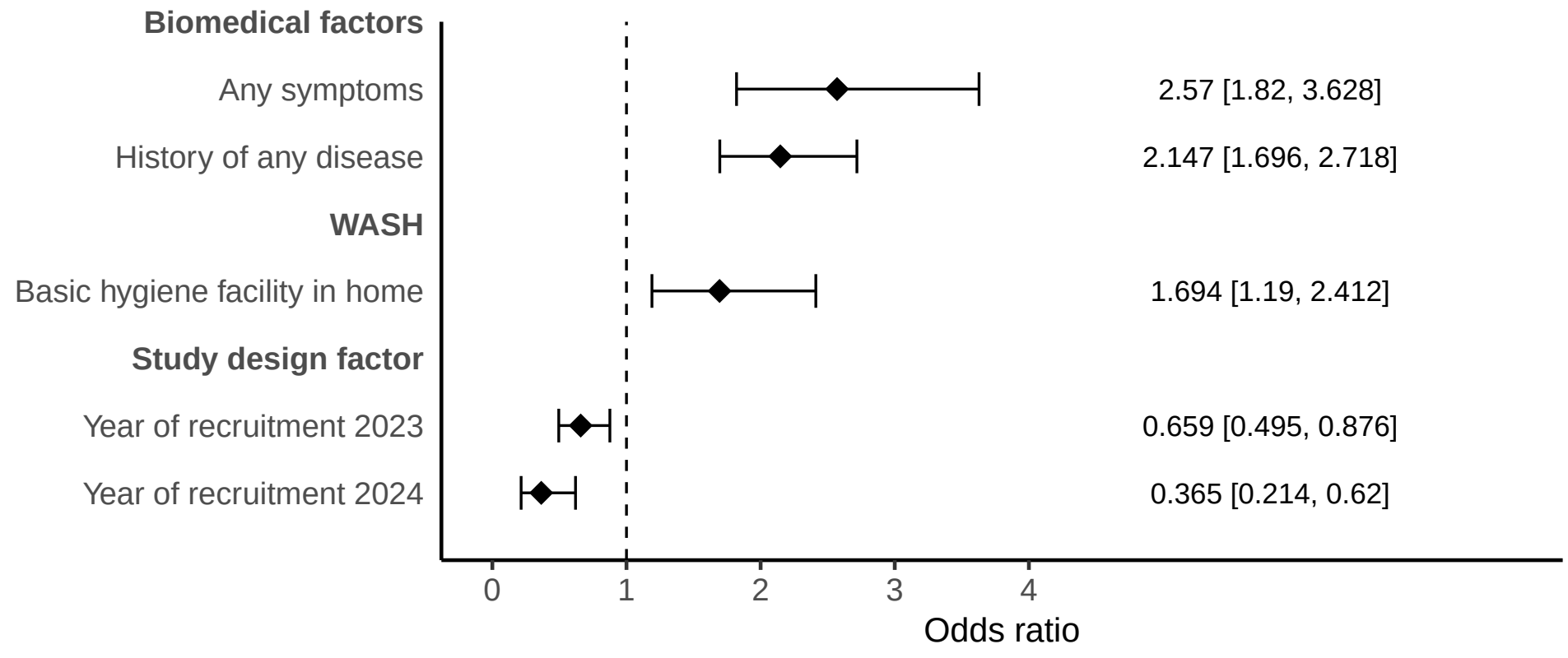

Fig. S2 Model for any care sought for adults. Multilevel model for any care sought for individuals aged 20 and above ($n = 3780$) with random effects at the village and HH levels. VIFs<10 for all covariates, and AUC for 10-fold cross-validation was 0.656.

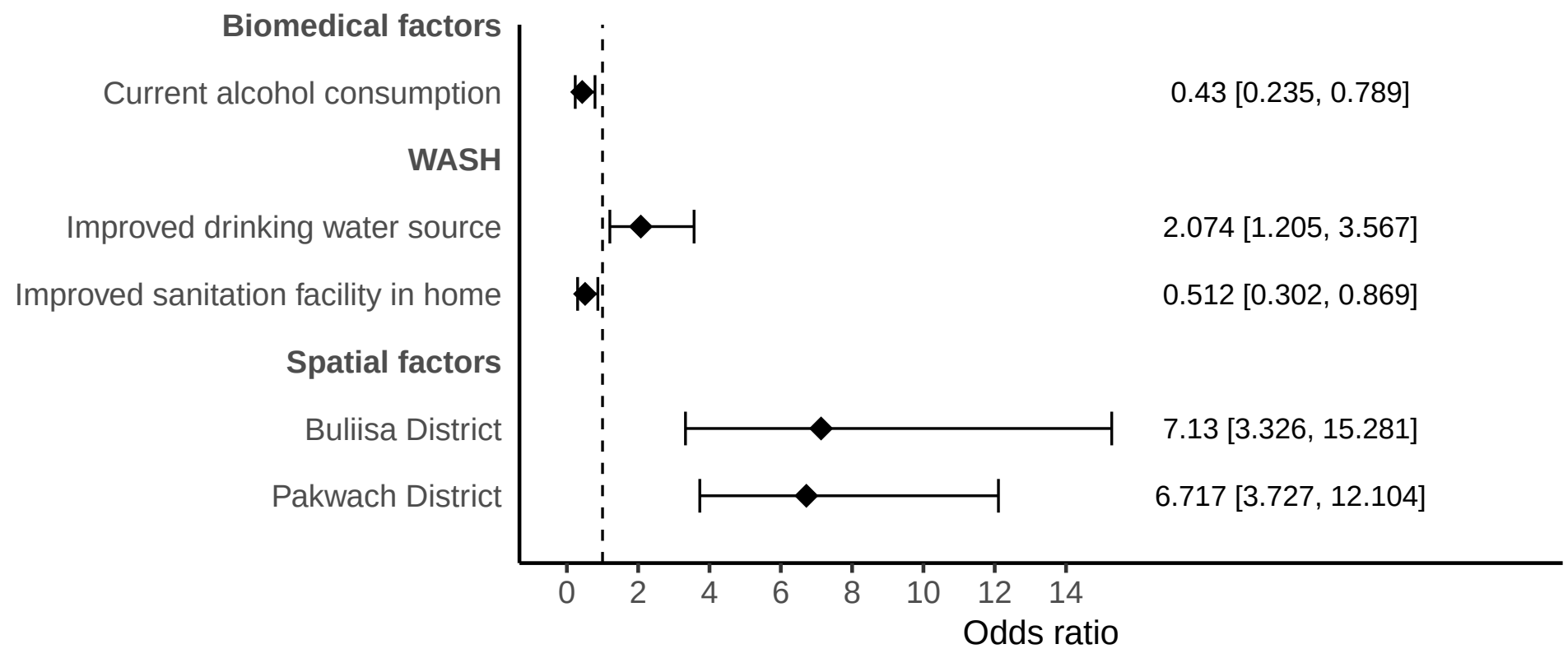

Fig. S3 Model for type of care sought for adults. Logistic regression model for the type of care sought for individuals aged 20 and above who sought care ($n = 420$) with 95% CIs calculated using clustered standard errors at the HH level. VIFs<10 for all covariates, and AUC for 10-fold cross-validation was 0.745.

Table S1 Self-reported medical history.

	Total	Care seeking	Not care seeking
	(N=8038)	(N=659)	(N=7379)
**HISTORY OF INFECTIOUS DISEASES**			
**Uncomplicated malaria**	1642 (20.4%)	216 (32.8%)	1426 (19.3%)
**Complicated malaria**	507 (6.3%)	84 (12.7%)	423 (5.7%)
**Encephalitis**	108 (1.3%)	15 (2.3%)	93 (1.3%)
**Pneumonia**	207 (2.6%)	36 (5.5%)	171 (2.3%)
**Pertussis**	679 (8.4%)	73 (11.1%)	606 (8.2%)
**Scabies**	135 (1.7%)	19 (2.9%)	116 (1.6%)
**Tetanus**	33 (0.4%)	2 (0.3%)	31 (0.4%)
**Typhoid**	1306 (16.2%)	165 (25.0%)	1141 (15.5%)
**Cholera**	625 (7.8%)	63 (9.6%)	562 (7.6%)
**Dysentery**	339 (4.2%)	45 (6.8%)	294 (4.0%)
**Diarrhoea**	608 (7.6%)	65 (9.9%)	543 (7.4%)
**Tuberculosis**	65 (0.8%)	17 (2.6%)	48 (0.7%)
**Meningitis**	33 (0.4%)	7 (1.1%)	26 (0.4%)
**Hepatitis B**	41 (0.5%)	5 (0.8%)	36 (0.5%)
**Hepatitis C**	17 (0.2%)	2 (0.3%)	15 (0.2%)
**Measles**	45 (0.6%)	12 (1.8%)	33 (0.4%)
**HIV**	127 (1.6%)	20 (3.0%)	107 (1.5%)
**Sepsis**	62 (0.8%)	6 (0.9%)	56 (0.8%)
**Leprosy**	28 (0.3%)	2 (0.3%)	26 (0.4%)
**Brucellosis**	27 (0.3%)	4 (0.6%)	23 (0.3%)
**Gut worms**	952 (11.8%)	129 (19.6%)	823 (11.2%)
**Bilharzia**	330 (4.1%)	58 (8.8%)	272 (3.7%)
**Trachoma**	285 (3.5%)	47 (7.1%)	238 (3.2%)
**Hemorrhagic fever**	49 (0.6%)	6 (0.9%)	43 (0.6%)
**COVID**	19 (0.2%)	2 (0.3%)	17 (0.2%)
**HISTORY OF NON-COMMUNICABLE DISEASES**			
**Epilepsy**	55 (0.7%)	8 (1.2%)	47 (0.6%)
**Sickle cell disease**	49 (0.6%)	9 (1.4%)	40 (0.5%)
**Anaemia**	93 (1.2%)	14 (2.1%)	79 (1.1%)
**Liver disease**	180 (2.2%)	25 (3.8%)	155 (2.1%)
**Kidney disease**	58 (0.7%)	13 (2.0%)	45 (0.6%)
**Asthma**	152 (1.9%)	19 (2.9%)	133 (1.8%)
**Eczema**	752 (9.4%)	68 (10.3%)	684 (9.3%)
**Cancer**	41 (0.5%)	6 (0.9%)	35 (0.5%)
**Depression**	102 (1.3%)	22 (3.3%)	80 (1.1%)
**Anxiety**	82 (1.0%)	13 (2.0%)	69 (0.9%)
**Psychosis**	17 (0.2%)	3 (0.5%)	14 (0.2%)

Table S2 Prevalence of past fever and diarrhoea among symptomatic participants across the study districts.

Past symptom reported	Total	Pakwach	Buliisa	Mayuge	*p*-value[a]
	(N=285)	(N=224)	(N=50)	(N=11)	
Fever	16 (5.6%)	11 (4.9%)	5 (10%)	0 (0.0%)	0.52
Diarrhoea	27 (9.5%)	20 (8.9%)	7 (14%)	0 (0.0%)	0.82

[a] *p*-values were calculated using Rao-Scott second-order corrected chi-square tests to account for HH and village-level clustering.

Table S3 Care-seeking behaviour across study districts.

	Overall	Pakwach	Buliisa	Mayuge	*p*-value[a]
**Individual level**	(N=8038)	(N=3313)	(N=2455)	(N=2270)	
Care seeking	659 (8.2%)	316 (9.5%)	151 (6.2%)	192 (8.5%)	0.258
Govt. health centre[b]	(n=659)	(n=316)	(n=151)	(n=192)	
	445 (67.5%)	254 (80.4%)	123 (81.5%)	68 (35.4%)	<0.001
**Usual source of care by HHs**	(N=2191)	(N=897)	(N=745)	(N=549)	
Govt. health centre	1858 (84.8%)	800 (89.2%)	689 (92.5%)	369 (67.2%)	<0.001
Private clinic	252 (11.5%)	28 (3.1%)	49 (6.6%)	175 (31.9%)	
Local medicine shop	37 (1.7%)	27 (3.0%)	6 (0.8%)	4 (0.7%)	
VHT	44 (2%)	42 (4.7%)	1 (0.1%)	1 (0.2%)	

[a] *p*-values were calculated using Rao-Scott second-order corrected chi-square tests to account for HH and village-level clustering.
[b] This is among participants who reported seeking care in the month before recruitment.

Table S4 HH characteristics.

	Total	HH health care other	HH health care PHC
	(N=2191)	(N=289)	(N=1902)
**HH HEALTH**			
**History of IDs**	1470 (67.1%)	194 (67.1%)	1276 (67.1%)
**History of NCDs**	895 (40.8%)	103 (35.6%)	792 (41.6%)
**History of any disease**	1601 (73.1%)	213 (73.7%)	1388 (73.0%)
**SOCIODEMOGAPHICS**			
**Highest level of education attained of HH head**			
Mean (SD)	4.66 (3.31)	4.90 (3.20)	4.62 (3.33)
**Occupation of HH head**			
Farmer	571 (26.1%)	85 (29.4%)	486 (25.6%)
Fisherman	541 (24.7%)	81 (28.0%)	460 (24.2%)
Fishmonger	107 (4.9%)	9 (3.1%)	98 (5.2%)
None/Other	972 (44.4%)	114 (39.4%)	858 (45.1%)
**Home quality score**			
Mean (SD)	5.76 (3.44)	7.37 (3.58)	5.52 (3.35)
**HH social status**	257 (11.7%)	24 (8.3%)	233 (12.3%)
**Number of individuals in HH**			
Mean (SD)	3.66 (1.46)	3.92 (1.40)	3.62 (1.46)
**Deaths in HH (past 3 yrs)**	161 (7.3%)	19 (6.6%)	142 (7.5%)
**Years HH has lived in village**			
Mean (SD)	20.0 (15.6)	20.3 (14.4)	20.0 (15.8)
**Home owned**	1898 (86.6%)	252 (87.2%)	1646 (86.5%)
**Number of rooms**			
Mean (SD)	2.14 (1.17)	2.17 (1.06)	2.14 (1.18)
**WATER, SANITATION AND HYGIENE (WASH)**			
**Improved drinking water source**	1184 (54.0%)	141 (48.8%)	1043 (54.8%)
**HH treats drinking water**	489 (22.3%)	74 (25.6%)	415 (21.8%)
**Improved sanitation facility in home**	1253 (57.2%)	192 (66.4%)	1061 (55.8%)
**Basic hygiene facility in home**	183 (8.4%)	32 (11.1%)	151 (7.9%)
**SPATIAL FACTORS**			
**Min. dist. (km) to drug shop**			
Mean (SD)	1.11 (1.22)	0.618 (0.912)	1.19 (1.24)
**Min. dist. (km) to gov't. health centre**			
Mean (SD)	2.62 (1.60)	3.34 (1.68)	2.51 (1.56)
**District**			
Buliisa	745 (34.0%)	55 (19.0%)	690 (36.3%)
Mayuge	549 (25.1%)	179 (61.9%)	370 (19.5%)
Pakwach	897 (40.9%)	55 (19.0%)	842 (44.3%)
**STUDY DESIGN FACTOR**			
**Year of recruitment**			
2022	1459 (66.6%)	267 (92.4%)	1192 (62.7%)
2023	493 (22.5%)	11 (3.8%)	482 (25.3%)
2024	239 (10.9%)	11 (3.8%)	228 (12.0%)

Table S5 Characteristics of adults.

	Total	Care seeking	Not care seeking
	(N=3780)	(N=420)	(N=3360)
**BIOMEDICAL FACTORS**			
**Any symptoms**	232 (6.1%)	59 (14.0%)	173 (5.1%)
**IDs for other HH members**	2299 (60.8%)	295 (70.2%)	2004 (59.6%)
**NCDs for other HH members**	1210 (32.0%)	151 (36.0%)	1059 (31.5%)
**History of IDs**	1880 (49.7%)	265 (63.1%)	1615 (48.1%)
**History of NCDs**	944 (25.0%)	151 (36.0%)	793 (23.6%)
**History of any disease**	2094 (55.4%)	305 (72.6%)	1789 (53.2%)
**Current alcohol consumption**	616 (16.3%)	76 (18.1%)	540 (16.1%)
**Current smoker**	445 (11.8%)	51 (12.1%)	394 (11.7%)
**SOCIODEMOGAPHICS**			
**Age**			
Mean (SD)	38.9 (13.4)	40.9 (14.5)	38.7 (13.2)
**Sex - Female**	2131 (56.4%)	256 (61.0%)	1875 (55.8%)
**Majority tribe**	2838 (75.1%)	331 (78.8%)	2507 (74.6%)
**Majority religion**	2796 (74.0%)	312 (74.3%)	2484 (73.9%)
**Highest level of education attained**			
Mean (SD)	4.57 (3.26)	4.41 (3.23)	4.59 (3.26)
**Occupation**			
Farmer	1108 (29.3%)	137 (32.6%)	971 (28.9%)
Fisherman	589 (15.6%)	58 (13.8%)	531 (15.8%)
Fishmonger	218 (5.8%)	30 (7.1%)	188 (5.6%)
None/Other	1865 (49.3%)	195 (46.4%)	1670 (49.7%)
**Home quality score**			
Mean (SD)	5.88 (3.49)	5.90 (3.55)	5.88 (3.48)
**HH social status**	498 (13.2%)	50 (11.9%)	448 (13.3%)
**Number of individuals in HH**			
Mean (SD)	3.94 (1.56)	3.77 (1.66)	3.96 (1.54)
**Deaths in HH (past 3 yrs)**	299 (7.9%)	52 (12.4%)	247 (7.4%)
**Years HH has lived in village**			
Mean (SD)	20.2 (15.4)	21.5 (15.6)	20.0 (15.4)
**Home owned**	3295 (87.2%)	363 (86.4%)	2932 (87.3%)
**Number of rooms**			
Mean (SD)	2.21 (1.22)	2.25 (1.44)	2.21 (1.18)
**WATER, SANITATION AND HYGIENE (WASH)**			
**Improved drinking water source**	2059 (54.5%)	253 (60.2%)	1806 (53.8%)
**HH treats drinking water**	863 (22.8%)	120 (28.6%)	743 (22.1%)
**Improved sanitation facility in home**	2227 (58.9%)	227 (54.0%)	2000 (59.5%)
**Basic hygiene facility in home**	346 (9.2%)	61 (14.5%)	285 (8.5%)
**SPATIAL FACTORS**			
**Min. dist. (km) to drug shop**			
Mean (SD)	1.09 (1.20)	1.26 (1.23)	1.07 (1.19)
**Min. dist. (km) to gov't. health centre**			
Mean (SD)	2.65 (1.62)	2.63 (1.63)	2.65 (1.61)
**District**			
Buliisa	1225 (32.4%)	94 (22.4%)	1131 (33.7%)
Mayuge	1004 (26.6%)	116 (27.6%)	888 (26.4%)
Pakwach	1551 (41.0%)	210 (50.0%)	1341 (39.9%)
**STUDY DESIGN FACTOR**			
**Year of recruitment**			
2022	2538 (67.1%)	324 (77.1%)	2214 (65.9%)
2023	823 (21.8%)	78 (18.6%)	745 (22.2%)
2024	419 (11.1%)	18 (4.3%)	401 (11.9%)

Table S6 HH distances to PHCs and medicine shops by district.

District	Number of HHs	Median distance to PHC (km)	Percentage of HHs within 5km of a PHC	Median distance to medicine shop (km)	Percentage of HHs within 5km of a medicine shop
Pakwach	897	2.41 (1.75-4.57)	87.1	0.9 (0.33-2.23)	100
Buliisa	745	2.5 (0.6-3.02)	100	0.34 (0.13-3.04)	98.4
Mayuge	549	2.05 (1.26-4.86)	75.2	0.18 (0.08-0.4)	100

Table S7 Reported final diagnoses from government health centres. The categories are not mutually exclusive as some individuals may have had more than one diagnosis. Percentages were computed against a total of 341 individuals who had non-missing information on final diagnoses from government health centres.

Symptom	Count	Percentage
**Malaria**	195	57.18
**Other**	25	7.33
**Pregnancy-related**	17	4.99
**Headache**	9	2.64
**Ulcer**	8	2.35
**Cough**	7	2.05
**HIV**	7	2.05
**Typhoid**	5	1.47
**Abdominal pain**	5	1.47
**Flu**	4	1.17
**Epilepsy**	3	0.88
**AIDS**	3	0.88
**Asthma**	3	0.88
**Tuberculosis**	3	0.88
**Worms**	3	0.88
**Dental problem**	2	0.59
**Kidney failure**	2	0.59
**Diabetes**	2	0.59
**Fever**	2	0.59
**Stomach pain**	2	0.59
**Allergy**	2	0.59
**Sickle cell**	2	0.59
**Syphilis**	2	0.59
**Traumatic brain injury**	1	0.29
**Yellow fever**	1	0.29
**Appendicitis**	1	0.29
**Splenomegaly**	1	0.29
**Mental disorder**	1	0.29
**Vomiting blood**	1	0.29
**Anaemia**	1	0.29
**Hepatitis B**	1	0.29
**Thoracic disease**	1	0.29
**Ophthalmic disease**	1	0.29
**Anemia**	1	0.29
**Dysentry**	1	0.29
**Respiratory tract infections**	1	0.29
**Wounds**	1	0.29
**Skin rashes**	1	0.29
**Back pain**	1	0.29
**Hypertension**	1	0.29
**Heart attack**	1	0.29
**Bilharzia**	1	0.29
**Haematuria**	1	0.29
**Intestinal operation**	1	0.29
**Measles**	1	0.29
**Hernia**	1	0.29
**Liver enlargement**	1	0.29
**Spleen enlargement**	1	0.29
**Uterus problem**	1	0.29
**General health check**	1	0.29
**Chest pain**	1	0.29
