# Supplementary material for: Medical, socioeconomic, and geographic disparities in primary health care access and utilization: a population-based study of 8038 individuals aged one year and older in rural Uganda
Source: BMC Med. 2026 Jul 21;24:569. doi: 10.1186/s12916-026-05061-x (PMC13682141; doi:10.1186/s12916-026-05061-x)
Supplement: Supplementary file 2 [file 12916_2026_5061_MOESM2_ESM.docx]

STROBE Statement—checklist of items that should be included in reports of observational studies

|  | Item No. | Recommendation | Page  No. | Relevant text from manuscript |
| --- | --- | --- | --- | --- |
| **Title and abstract** | 1 | (*a*) Indicate the study’s design with a commonly used term in the title or the abstract | 1 | Population-based study |
|  |  | (*b*) Provide in the abstract an informative and balanced summary of what was done and what was found | 1-2 | Primary health care access. Seeking any care. |
| Introduction | | | |  |
| Background/rationale | 2 | Explain the scientific background and rationale for the investigation being reported | 2-3 | Access to PHC—one component of the choice of usual care—has primarily relied on studies that focus on geographic barriers. Need for studies that incorporate the health knowledge a patient has of their own medical history, as well as the knowledge a patient has of the medical histories of other household (HH) members. |
| Objectives | 3 | State specific objectives, including any prespecified hypotheses | 3 | Predict PHC usage for individuals aged one year and older from 52 rural Ugandan communities. |
| Methods | | | |  |
| Study design | 4 | Present key elements of study design early in the paper | 4 | Prospective, community-based cohort set up to examine the development and progression of liver fibrosis related to schistosomiasis. |
| Setting | 5 | Describe the setting, locations, and relevant dates, including periods of recruitment, exposure, follow-up, and data collection | 3-4 | This study was conducted in Mayuge, Buliisa, and Pakwach Districts in Uganda along Lake Victoria, Lake Albert, and the Nile River, respectively. Three rounds of recruitment from 2022 to 2024 were completed during January to February of each year. |
| Participants | 6 | (*a*) *Cohort study*—Give the eligibility criteria, and the sources and methods of selection of participants. Describe methods of follow-up  *Case-control study*—Give the eligibility criteria, and the sources and methods of case ascertainment and control selection. Give the rationale for the choice of cases and controls  *Cross-sectional study*—Give the eligibility criteria, and the sources and methods of selection of participants | 4 | Approximately forty HHs were systematically randomly sampled using village registers ordered by year of first residence from 52 villages; details are provided elsewhere. |
|  |  | (*b*) *Cohort study*—For matched studies, give matching criteria and number of exposed and unexposed  *Case-control study*—For matched studies, give matching criteria and the number of controls per case |  |  |
| Variables | 7 | Clearly define all outcomes, exposures, predictors, potential confounders, and effect modifiers. Give diagnostic criteria, if applicable | 4-6 | Health access and utilisation outcomes of any care sought, and the main type of care sought within the one month preceding study recruitment. Medical histories with no restricted timeline and clinical symptoms experienced in the month preceding recruitment. Clinical symptoms were recorded as free text and grouped. Indicators related to other HH members were recorded. Demographic, socioeconomic, and water, sanitation, and hygiene (WASH) data were collected at the individual or HH level. For spatial factors, the minimum distances from HHs to drug shops and government health centres were calculated. Travel time was recorded for participants who had visited a government PHC centre. |
| Data sources/ measurement | 8* | For each variable of interest, give sources of data and details of methods of assessment (measurement). Describe comparability of assessment methods if there is more than one group | 4-5 | An electronic pre-validated survey programmed in Open Data Kit Collect. self-reported past diagnoses for both infectious diseases (IDs) using validated modules from World Health Surveys, and for non-communicable diseases (NCDs) using modules on hypertension, stroke, cholesterol, and diabetes from World Health Organization (WHO) STEPwise approach (STEPS) surveys. |
| Bias | 9 | Describe any efforts to address potential sources of bias | 15 | One of the study limitations is our focus on care-seeking only in the one month before recruitment. While this was intended to minimise recall bias, we acknowledge this shorter timeframe may potentially lead to an underreporting of overall PHC use. |
| Study size | 10 | Explain how the study size was arrived at | 4 | Approximately forty HHs were systematically randomly sampled using village registers ordered by year of first residence from 52 villages; details are provided elsewhere. |

Continued on next page

| Quantitative variables | 11 | Explain how quantitative variables were handled in the analyses. If applicable, describe which groupings were chosen and why | 4-6 | Coded as binary indicators and measured at the individual level. A binary indicator was generated that was equal to one if a participant had experienced any symptoms in the month preceding recruitment into the cohort. For spatial factors, the minimum distances from HHs to drug shops and government health centres were calculated by computing the Euclidean distance between coordinate points on the World Geodetic System 84 ellipsoid. |
| --- | --- | --- | --- | --- |
| Statistical methods | 12 | (*a*) Describe all statistical methods, including those used to control for confounding | 7 | Descriptive comparisons of past symptoms and care-seeking behaviour across districts were assessed using Rao-Scott second-order corrected chi-squared tests. Multilevel logistic regression was run for the individual-level outcomes of any care sought with random effects for HH and village. Intraclass clustering coefficients were calculated for empty and adjusted multilevel models. Ordinary logistic regression with clustered standard errors at the HH level was run for the outcome of the type of care which was a sub-group analysis of only participants who had sought care. Covariate selection was done for each model by finding the best fit parsimonious model, using backward selection and the lowest Bayesian Information Criterion. For regression models where the year of recruitment variable was significant, floating absolute risks were computed. |
|  |  | (*b*) Describe any methods used to examine subgroups and interactions | 6 | For the usual source of care, logistic regressions were run at the HH level using only covariates measured at the HH level. |
|  |  | (*c*) Explain how missing data were addressed | 6 | Two adults missing STIs and NCDs, and two participants with incomplete HH information. (excluded) |
|  |  | (*d*) *Cohort study*—If applicable, explain how loss to follow-up was addressed  *Case-control study*—If applicable, explain how matching of cases and controls was addressed  *Cross-sectional study*—If applicable, describe analytical methods taking account of sampling strategy |  |  |
|  |  | (*e*) Describe any sensitivity analyses | 7 | Each model was checked for multicollinearity by calculating the variance inflation factors (VIFs). |
| Results | | | | |
| Participants | 13* | (a) Report numbers of individuals at each stage of study—eg numbers potentially eligible, examined for eligibility, confirmed eligible, included in the study, completing follow-up, and analysed | 7 | 8038 participants recruited in SchistoTrack. |
|  |  | (b) Give reasons for non-participation at each stage |  |  |
|  |  | (c) Consider use of a flow diagram |  |  |
| Descriptive data | 14* | (a) Give characteristics of study participants (eg demographic, clinical, social) and information on exposures and potential confounders | 12 | Table 1 |
|  |  | (b) Indicate number of participants with missing data for each variable of interest |  |  |
|  |  | (c) *Cohort study*—Summarise follow-up time (eg, average and total amount) |  |  |
| Outcome data | 15* | *Cohort study*—Report numbers of outcome events or summary measures over time | 7 | There were 8.2% (659/8038) participants who sought any medical care in the month before recruitment. |
|  |  | *Case-control study—*Report numbers in each exposure category, or summary measures of exposure |  |  |
|  |  | *Cross-sectional study—*Report numbers of outcome events or summary measures |  |  |
| Main results | 16 | (*a*) Give unadjusted estimates and, if applicable, confounder-adjusted estimates and their precision (eg, 95% confidence interval). Make clear which confounders were adjusted for and why they were included | 9-10 | Figure 1 shows the multilevel model for any care sought for all participants with random effects at the village and HH levels. |
|  |  | (*b*) Report category boundaries when continuous variables were categorized |  |  |
|  |  | (*c*) If relevant, consider translating estimates of relative risk into absolute risk for a meaningful time period | 9 | Differences between participants in 2022 and 2023 being borderline significant based on the FAR estimates. |

Continued on next page

| Other analyses | 17 | Report other analyses done—eg analyses of subgroups and interactions, and sensitivity analyses | 9, 11 | Figure 3 shows the model predicting the usual source of care (government health centre and VHTs versus private health care). |
| --- | --- | --- | --- | --- |
| Discussion | | | | |
| Key results | 18 | Summarise key results with reference to study objectives | 10-14 | The usage of PHC services was low at 67.5% of participants who sought care within the month preceding the study, and was influenced by several complex medical and social factors beyond conventionally studied barriers of the distance of a HH to a PHC centre. |
| Limitations | 19 | Discuss limitations of the study, taking into account sources of potential bias or imprecision. Discuss both direction and magnitude of any potential bias | 15 | Some limitations include that we focus on the main source of care either within the past month or usual source used by the HH. |
| Interpretation | 20 | Give a cautious overall interpretation of results considering objectives, limitations, multiplicity of analyses, results from similar studies, and other relevant evidence | 14-16 | We have shown that the access to health care and usage of PHC in rural sub-African settings is determined by a complex set of factors. |
| Generalisability | 21 | Discuss the generalisability (external validity) of the study results | 16 | If replicated in other countries, we anticipate our findings can guide strategies for improving the access to and utilisation of PHC services in sub-Saharan Africa. |
| Other information | |  | | |
| Funding | 22 | Give the source of funding and the role of the funders for the present study and, if applicable, for the original study on which the present article is based | 16 | GFC received funding from the Wellcome Trust Institutional Strategic Support Fund (204826/Z/16/Z), Robertson Foundation Fellowship (Grant/Award Number: Not Applicable), and UKRI EPSRC Award (EP/X021793/1). |

*Give information separately for cases and controls in case-control studies and, if applicable, for exposed and unexposed groups in cohort and cross-sectional studies.

**Note:** An Explanation and Elaboration article discusses each checklist item and gives methodological background and published examples of transparent reporting. The STROBE checklist is best used in conjunction with this article (freely available on the Web sites of PLoS Medicine at http://www.plosmedicine.org/, Annals of Internal Medicine at http://www.annals.org/, and Epidemiology at http://www.epidem.com/). Information on the STROBE Initiative is available at www.strobe-statement.org.
